# Dentin Sialoprotein/Phosphophoryn (DSP/PP) as Bio-Inductive Materials for Direct Pulp Capping

**DOI:** 10.3390/polym14173656

**Published:** 2022-09-03

**Authors:** Shu-Fen Chuang, Yu-Hsuan Chen, Peter X. Ma, Helena H. Ritchie

**Affiliations:** 1 School of Dentistry and Institute of Oral Medicine, National Cheng Kung University, No. 1 Universal Road, Tainan 70101, Taiwan; 2 Department of Stomatology, National Cheng Kung University Hospital, 138 ShengLi Road, Tainan 70403, Taiwan; 3 Department of Biologic and Materials Sciences, School of Dentistry, University of Michigan, 1011 North University Avenue, Ann Arbor, MI 48109-1078, USA; 4 Department of Cariology, Restorative Sciences and Endodontics, School of Dentistry, University of Michigan, 1011 North University Avenue, Ann Arbor, MI 48109-1078, USA

**Keywords:** pulp capping, regeneration, phosphophoryn, dentin sialoprotein, RNA interference, reparative dentin

## Abstract

Conventional direct pulp capping, such as calcium hydroxide (Ca(OH)_2_) or silicate products, usually induces an inflammatory reaction to provoke pulp regeneration. Phosphophoryn (PP) and dentin sialoprotein (DSP), the two most abundant non-collagenous proteins in the dentin matrix, are responsible for dentin mineralization, pulp cell migration, and differentiation. Here we examined the PP and combined DSP/PP as bio-inductive pulp capping materials by in vitro and in vivo tests. Firstly, the effects of the PP dose on pulp cell migration and matrix protein expression were examined by an agarose bead test. Secondly, the role of recombinant DSP (recDSP) and recDSP/PP on stimulating DSP-PP transcript expression was examined by RT-PCR. DSPP mRNA was also knocked down by RNA interference (RNAi) to examine their functions on dentin matrix mineralization. Finally, we used ferret animal models to test PP and recDSP/PP acting as capping agents on in vivo pulp responses and reparative dentin formation. The result showed that intermediate-dose PP was the most effective to enhance cell migration and differentiation. RecDSP/PP strongly enhanced the DSP-PP transcript expression, while inhibition of DSPP mRNA expression by siRNAs partially or completely affected dental pulp cell mineralization. The in vivo results showed that intermediate-dose PP and recDSP/PP proteins induced less pulp inflammation and promoted reparative dentin formation. Contrarily, conventional calcium hydroxide induced severe pulp inflammation. With these findings, DSP and PP could serve as capping agents for pulp capping therapy.

## 1. Introduction

In clinical cases of extensive dental caries, pulp tissue is endangered by the toxins formed by the cariogenic microorganisms, the iatrogenic mechanical trauma during removal of caries and cavity preparations, and the chemical irritants from the restorative materials. After cleaning the carious and softened tooth substances, pulp tissue exposure may result. The dental pulp chamber is almost fully encapsulated by hard dentin, and the only connection with the peripheral blood and lymph circulation is through the tiny apical foramen at the root apexes [1]. The limited linkage with outer tissues makes the dental pulp vulnerable to inflammation and necrotic change [2]. Dentists consider protecting the pulp tissue, maintaining its vitality, and supporting the physiology and functions of the dentino-pulpal complex as the primary goal.

The conventional pulp capping is performed by using protective materials such as calcium hydroxide (Ca(OH)_2_) or other mineral compounds as the thermal insulator or a barrier from the chemical irritations and the microbial attacks. Calcium hydroxide has been advocated as the gold standard for direct pulp capping for several decades due to its antibacterial property and effectiveness in stimulating reparative dentin formation [3,4,5]. However, its high solubility, lack of adhesion, and poor mechanical properties lead to a poor seal of the pulp chamber [6]. In addition, calcium hydroxide causes strong stimuli to the pulp, and hence the replacement odontoblasts are not well-differentiated and “tunnel defects” are formed in the reparative dentin [7,8,9]. With the advance of vital pulp therapy, some bioactive materials are developed to initiate the repair process and promote pulp regeneration and revascularization. Functional capping materials such as ProRoot MTA(MTA; Dentsply Tulsa Dental), Biodentine (Septodont), or other calcium silicates have been approved to activate the damaged odontoblasts or stimulate the multipotent progenitor cells in the pulp core to differentiate into the replacement cells [4,5,10]. These materials may also increase expression of transforming growth factor-beta 1 (TGF-β1) in human pulp cells and induce mineralization foci in a tooth culture model [11,12]. Investigations have shown their efficacy in maintaining pulp vitality and inducing pulp regeneration through modulating a critical balance between inflammation and regeneration [13]. Almost all these capping materials have cytotoxicity with human mesenchymal cells, which stimulates odontoblastic differentiation and mineralization through enhancing DSPP and DMP1 mRNA expression [14,15]. However, persisting inflammation is particularly detrimental to pulp tissue in such an inaccessible and unyielding environment of pulp chamber and might eventually lead to pulp necrosis [13,16]. Accordingly, high clinical success rates of 65%–90% for direct pulp capping may be achieved within 1–2 years [3,17,18] but fluctuate between 56% and 81% after 4–5 years [19,20,21].

Apart from synthetic materials, the use of growth factors and/or growth factor-embedded materials for the vital pulp therapy are a new strategy [22]. During primary dentinogenesis, growth factors and bioactive molecules are secreted by odontoblasts and are incorporated within the dentine extracellular matrix [23]. Growth factors, including TGF-β1, insulin-like growth factor I and II (IGF-I and II), bone morphogenetic protein (BMP), vascular endothelial growth factor (VEGF), and pro- and anti-inflammatory cytokines, including IL-1α, IL-1β, IL-4, IL-6, IL-8, and IL-12, have also been reported as the signaling molecules that control and regulate cellular events involved in the repairing process [24,25,26]. Although the presentation and function of growth factors and cytokines during the pulp regeneration have been identified, they are mostly considered as sequestered and released by therapeutic materials. For the injured pulp, creating a favorable environment to maintain the physiology is the prerequisite. Functional capping materials and bioactive molecules have the potential to initiate the regeneration events and promote fast repair, including the chemotactic migration, cell differentiation, proliferation, and deposition of mineral matrix, which is preferred for reparative dentinogenesis.

During the tooth development, the reciprocal epithelial–mesenchymal interactions are critical for odontoblast differentiation and dentin formation. Phosphophoryn (PP) and dentin sialoprotein (DSP) are two major dentinal non-collagenous extracellular matrixes which are derived from dentinsialoprotein-phosphophoryn (DSP-PP; also termed dentinsialophosphoprotein, DSPP) gene. PP plays an important role in the transition of predentin to dentin, which is necessary for the formation of mineralized teeth. To initiate this process, PP generates insoluble mineral aggregates by binding large amounts of calcium ions [27,28] and subsequently interacting with the “e” band of collagen to form a stable support for apatite crystallization [29]. Finally, the association of PP with collagen creates a nucleation site where mineralization can occur. DSP is the second most abundant noncollagenous protein in dentin. DSP may act as a structural protein or be involved in modulating hydroxyapatite formation. Immunolocalization experiments showed that DSP is expressed in odontoblasts that are actively participating in dentin mineralization [30].

The transient expression of DSP protein and DSP-PP transcripts, first in the preameloblasts and next in both preameloblasts and young odontoblasts, suggest that these two proteins might be able to induce dental pulp cell migration, odontoblast differentiation, and subsequent dentin formation [30,31]. Our previous work has applied a new experimental model to examine the chemotactic activity of native PP to attract dental pulp cell migration and differentiation [32]. The results showed that PP at intermediate concentration exhibited the superiority in promoting proliferation and migration of dental pulp cells compared to the higher concentration. The effect of PP on the mineralization process was also concentration dependent, which incidentally corresponds to the “multifunctional” role of the dentin PP [33,34]. PP in low concentrations (0.01–1 μg/mL) promoted the hydroxyapatite formation but at a higher concentration (100 μg/mL) inhibited hydroxyapatite growth [33]. We also demonstrated that the combination of DSP and PP proteins (DSP/PP) could reduce cell proliferation and cell morphology as well as promote DSP-PP mRNA expression [32]. DSP/PP proteins are likely responsive to recapturing the odontoblast developmental processes, such as by promoting cell migration from dental pulp toward preameloblasts, expressing DSP-PP mRNA, and inducing cell differentiation to up-regulate DSP-PP transcript expression, eventually leading to reparative dentin formation. However, DSP/PP also reduce cell proliferation. In this regard, the effect of DSP/PP on dentin regeneration needs further inspection. Nevertheless, previous researchers hardly examined the effect of PP on modulating the behavior of odontoblasts and pulpal responses under the in vivo model.

Here we comprehensively evaluated PP at different doses and DSP/PP proteins serving as a bio-inductive material for direct pulp capping. At first, the effects of PP and recombinant (rec) DSP/PP proteins on affected cell proliferation, migration, and differentiation was examined by using dental pulp cell lines. The functions of PP and DSP/PP in promoting extracellular matrix synthesis and mineralization were evaluated in in vitro tissue culture. A ferret animal model was established to evaluate the in vivo effects of PP and DSP/PP as capping materials on pulp tissue health, and the dentinogenesis, with a specific aim to examine whether PP by itself can promote mineralization or whether a DSP/PP mixture can better promote this process.

## 2. Materials and Methods

### 2.1. Effect of PP Concentration on Proliferation and Migration

The examination of the chemotactic ability of PP to pulp cells (MRPC-1) refers to our established method [32]; 1% agarose sol was prepared by dissolving 30 μg dry agarose powder (Sigma-Aldrich, St. Louis, MO, USA) into 3 mL deionized water, heated to 100 °C for 10 s, then kept in a 60 °C water bath. Five groups of cell culture were examined: (1) control, cell culture without agarose gel; (2) PP(−), cell culture with a agarose bead without PP; (3) L-PP, cell culture with a agarose bead containing low dose (0.2 µg) PP; (4) I-PP, cell culture with a agarose bead containing intermediate dose (1 µg) PP; and (5) H-PP, cell culture with a agarose bead containing high does (5 µg) PP (n = 3). Native rat PP was generated from the transcription of rat DSP-PP_523_ referred to Marsh’s method [27,35]. For the latter three groups, pure PP were added into the agarose gel at 0.05, 0.25, and 1.25 μg/μL concentrations. In each group, 4 µL agarose sol were dispensed into the centers of 12-well culture plates and allowed to solidify. Therefore, the expected 0.2 μg, 1 μg, 5 μg PP were delivered respectively. 1.0 mL of cell suspension (2 × 10^5^ cells/mL) was inoculated in each well. The cell culture plates were incubated at 37 °C under 5% CO_2_: 95% air. The medium was changed every other day. At Days 2, 4 and 6, the cell morphology, their proliferation and their migration toward the gel were observed under an optical microscope (Nikon Eclipse TS100, Nikon Co., Tokyo, Japan).

### 2.2. Effect of PP on Col I and PP Expressions

The agarose gels are prepared and mixed with PP at different concentrations to detect collagen type I (Col I) and PP expression. The prepared agarose gels were dispensed into the 18-well culture plates to solidify as stated in Section 2.1. 1.0 mL of cell suspension of 2 × 10^5^ cells/mL concentration was inoculated and cultured at 37 °C for 6 days. On each of Days 2, 4, and 6, three culture wells in each group were used to detect the collagen type I (Col I) expression and another three wells for PP expression by using an immunohistochemistry (IHC) assay. The cultures were fixed with 4% formaldehyde (Sigma-Aldrich, St. Louis, MO, USA) for 1 h and then washed 3 times with PBS buffer. The primary antibody was prepared with either anti-Col I antibodies at 200× dilution or anti-PP antibodies at 100× dilution, in 1% blocking agent with maleic acid (Sigma-Aldrich, St. Louis, MO, USA). Diluted secondary biotinylated antibody was added and then shed and quenched of endogenous peroxidase activity. Finally, Vectastain Elite ABC reagent was added to the cultures and four drops of DAB stock solution (both from Vector laboratories, Newark, CA, USA) were added to each well for color observation.

### 2.3. Effect of Ascorbic Acid (AA), Recombinant Protein DSP_307_, DSP_370_, and DSP/PP_240_ Protein Mixture on PP Transcript Expression

Previously, we reported that M2H4 cells under different cultured treatments exhibited different cell morphological changes [32]. Here we examined whether the treatment of ascorbic acid (Sigma-Aldrich, St. Louis, MO, USA), recDSP_307,_ recDSP_370_ protein, and DSP/PP_240_ protein mixture affected their DSP-PP mRNA expression. Rat DSP-PP_240_ cDNA in a baculovirus expression vector pVL1392 was used to generate recombinant DSP_430_/PP_240_ (i.e., DSP protein with 430 amino acid residues and PP protein with 240 amino acid residues) proteins in the insect sf9 cell culture medium [36]. Recombinant DSP_307_ protein (i.e., DSP protein with 307 amino acid) and DSP_370_ protein (DSP protein with 370 amino acid residues) [36,37] were also generated in a baculovirus system and secreted into the insect culture medium.

M2H4 cells (8 × 10^5^ cells/T-25 flask) were cultured in 5 mL of specific growth medium (i.e., Eagle’s minimum essential medium containing 100 IU/mL penicillin and 100 µg/mL P/S and 10% FBS) for 8 days, with medium changed every other day. Once the cells reached 90% confluency, these cell cultures were divided into five groups and treated with different culture medium containing: (1) no ascorbic acid; (2) AA (50 µg/mL), AA(+); (3) AA and recombinant DSP_307_ protein(AA/DSP_307_); (4) AA and recombinant DSP_370_ protein mixture (AA/DSP_370_); and (5) AA and recombinant DSP_430_/PP_240_ protein mixture (AA/DSP/PP).

Cells were then harvested, and RNA was extracted. Reverse transcription-polymerase chain reaction (RT-PCR) was performed to determine the DSP-PP mRNA expression in each group. Briefly, the cells were extracted with Tri reagent (Sigma Chemical Co., St. Louis, MO, USA) to obtain total ribonucleic acid (RNA), which was then reverse transcribed with oligo deoxythymidylic acid (d[T]) primer and RT to generate complemental 3′DNA (cDNA) pools for further PCR analyses. A primer set comprising 5′-CGGTCCCTCAGTTAGTG-3′ (upper primer) and 5′-TACGTCCTCGCGTTCT-3′ (lower primer) was used to generate a 286-bp DNA fragment. Another primer set 5′-ACCACAGTCCATGCCATCAC-3′ and 5′-TCCACCACCCTGTTGCTGTA-3′ corresponding to a house keeping gene glyceraldehyde phosphate dehydrogenase (GADPH) was used as an internal control to generate a 450 bp PCR fragment. The PCR amplification was performed as follows: denaturation for 30 s at 94 °C, 30 s at 56 °C, and 72 °C for 30 cycles. The PCR products were run on 1.5% agarose gels containing 10 mg/mL ethidium bromide (Sigma-Aldrich, St. Louis, MO, USA) and photographed on an ultraviolet transilluminator.

### 2.4. Inhibition of DSPP mRNA Expressionby DSPP RNAi (RNA Interference)

Rat pulp MDPC-23 cells are well established dental pulp cells with characteristic of odontoblasts including high DSP and PP expression [38]. Here we used RNAi to reduce DSPP mRNA expression and evaluated whether inhibition of DSPP mRNA Expression affected the secretion of Col type I and mineralization by dental pulp cell mineralization.

MDPC-23 cells were seeded at 2.5 × 10^4^ cells/24 well and 2 h later, transfected with small interfering RNA (siRNA; DSPP3 siRNA and DSPP4 siRNA). After 24-h siRNA transfection, cells of the experimental group began to be treated with mineralization media (MM; 50 µg/mL ascorbic acid, 10 nM dexamethasone and 10 mM b-glycerophosphate; all reagents from Sigma-Aldrich, St. Louis, MO, USA) in α-MEM with 10% FBS, 2 mM glutamine (Gibco, Waltham, MA, USA) and P/S (50 units/mL) for 5 days. In another set of control group, the transfected cells were cultured in a non-mineralization media (α-MEM with 10% FBS, 2 mM glutamine and P/S 100 units/mL) for 5 days. On Day 3 and Day 5, cells were replaced with their respective medium or regular cultured medium. On Day 6, cells were harvested and fixed with 5% glutaraldehyde (Sigma-Aldrich, St. Louis, MO, USA) for 15 min, alizarin red (Sigma-Aldrich, St. Louis, MO, USA) staining. Positive siRNA, negative siRNA and mock transfections were included as controls. All RNAi experiments used Qiagen Flexitube siRNA Premix (Qiagen, Hilden, Germany). The areas of collagen matrix coverage and mineralization were measured by the Image J software (NIH, Baltimore, MD, USA) and transformed into the area percentages.

### 2.5. DSP/PP Proteins as Pulp Capping Agents in a Ferret Model

The effectiveness of low- and high-dose PP, and DSP/PP proteins as pulp capping materials on inducing pulp tissue repair and reactionary dentin formation was examined by using an animal study. The animal study was reviewed and approved by the University Committee on Use and Care of Animals (UCUCA) of the University of Michigan (Protocol #8614). Sixteen four-month-old healthy ferrets were selected for this study. Considering the tooth size, accessibility, and dentin thickness, the ferret canine was adapted as the experimental teeth to investigate the behaviors of these capping materials. PP of two doses (3.5 and 35 µg) was used in this experiment to represent its low and high loading doses.

The ferrets were sedated with ketamine/rompun ((Sigma-Aldrich, St. Louis, MO, USA). The following procedures were performed by an experienced operator. On each ferret, one cavity was prepared on each of four canine teeth with a sterilized high-speed handpiece under copious water irrigation. The final dimension of each cavity was approximately 2 mm in diameter, with 0.5 mm pulp exposed. Bleeding from pulp chamber was stopped by the compression of a sterilized cotton pellet. Five pulp capping treatments were included in this study as:

Group Col: One blank collagen membrane was placed on the pulp exposure site immediately after the bleeding was controlled. This group served as the negative control.

Group Dycal: The pulp exposure site was directly capped with the calcium hydroxide (Dycal, Dentsply, Milford, DE, USA). This group served as the positive control.

Group PP3.5: A collagen membrane preloaded with 3.5 μg rat native PP was used to cover the pulp exposure site. This group served as the low-dose PP experimental group.

Group PP35: The same procedure except that the collagen membrane preloaded with 35 μg rat native PP: This group served as the high-dose PP experimental group.

Group DSP/PP: The same procedure except that the collagen membrane preloaded with recDSP_430_/PP_240_ proteins.

For each ferret, four cavities were assigned to different treatments. The tooth number was 12 in group PP3.5 and 11 in the other groups.

**Figure 1:**
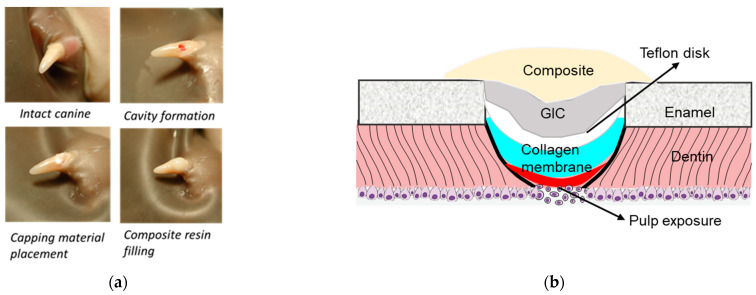
(**a**) The experimental procedures of pulp capping and coverage. (**b**) Diagram of the prepared cavity and capping materials. The pulp exposure site was covered by a capping material (Dycal, a blank collagen membrane, or a membrane containing PP or DSP/PP), followed by a Teflon disk, a thin layer of glass ionomer (GIC), and a resin composite.

After capping, the teeth were covered with a 2 mm diameter TeflonÒ disk and the cavity was sealed with a thin layer of a glass ionomer restorative material (Vitrebond, 3M ESPE, St. Paul, MN, USA) followed by a 20-s light curing. The GI filling was acid-etched for 15 s, a resin adhesive (Optibond Solo Plus, Kerr, Orange, CA, USA) was applied and light-cured for 10 s. Finally, the cavity was covered with a resin composite (Point 4 flowable composite, Kerr) and light-cured for 20 s. The experimental procedures and diagram are illustrated in Figure 1.

After treatment, the animals were checked daily for their health state. The ferrets were euthanized on Day 30. The animals were then perfused with 4% paraformaldehyde (Sigma-Aldrich, St. Louis, MO, USA). The restored teeth were extracted, stored in 4% paraformaldehyde, decalcified in a 10% EDTA (Sigma-Aldrich, St. Louis, MO, USA) solution, and finally embedded in paraffin (Sigma-Aldrich, St. Louis, MO, USA). The teeth were serially sectioned into 8-mm-thick specimens, mounted on glass slides, and then stained with hematoxylin-eosin (H&E) (Sigma-Aldrich, St. Louis, MO, USA). One assessor performed the observer-blind histologic examinations of the inflammatory reaction, hard tissue formation, and quality of reactionary dentin using the scoring systems by Liu et al. [39] and Long et al. [40] (Table 1).The statistical analyses of the pulp responses were conducted using a Kruskal–Wallis test, followed by a Mann–Whitney test for the pairwise comparisons.

## 3. Results

### 3.1. Effect of PP Concentration on Proliferation and Migration of MRPC-1 Cells

**Figure 2:**
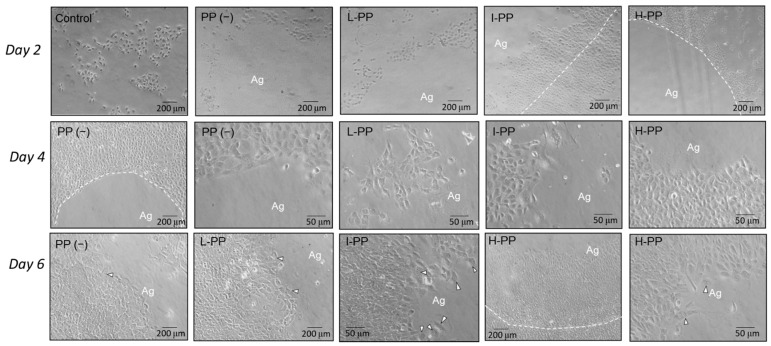
Histology of cell proliferation on Days 2, 4, and 6. On Day 2, cells in the control group scattered, while cells in the other groups colonized around the bead. Only cells in group I-PP intruded across the border of the agarose bead (indicated by the dash line). On Day 4, cells in PP(−) were still surround the agarose bead. L-PP, I-PP, and H-PP showed cells intruding into the beads. On Day 6, all groups show large squamoid cells intruding into agarose gel containing many granules. The activity of cell division is high (indicated by hollow arrowheads). Magnification at 40× or 200×.

On Day 2, the cultured cells in all groups appeared as spindle to squamoid shapes (Figure 2). The cells in control group were sparsely distributed. In groups with agarose beads, the amounts of cells increased with the PP doses. For groups L-PP and H-PP, cells migrated and located around the border of agarose bead. For group I-PP, the cells were intruded into the agarose beads.

On Day 4, the cell showed progressive proliferation and histology changes. In group PP(−), the cells maintained the spindle shape but had almost encircled the gel. The cells of L-PP and I-PP intruded into the bead which exhibited great cytoplasm and lamellipodium. The morphologic changes in group 4 were not obvious, and cell colonization was limited around the gel. On Day 6, the cells in group PP(−) turned into squamoid shape. In groups L-PP and I-PP, cells at the intruding front exhibited large lamellipodium, many granules, and a high tendency of cell division. In group H-PP, the migration activity and cell proliferation were inferior to groups L-PP and I-PP.

### 3.2. Effect of PP on Col I and PP Expressions

**Figure 3:**
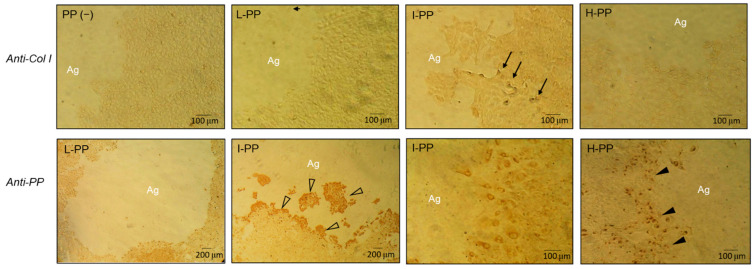
IHC stains of anti-Col I and anti-PP on Day 6. All cells showed insignificant anti-Col I activity except those in I-PP which exhibited antibody activity (indicated by arrows) at the invasion front. Contrarily, all groups showed anti-PP activity. I-PP present the highest immunohistochemistry stain and most stained cells (hollow arrowheads). In group H-PP, only single layer of cells surrounding the agarose present the anti-PP activity (indicated by solid arrowheads). Magnification at 100× and 200×.

The result of IHC assay is illustrated in Figure 3. Until Day 6, there was no significant anti-Col I activity shown in all of cell cultures. Minor antibody activity was only found in the cells at the invasion front in I-PP. Contrarily, the anti-PP activity was more significant. On day 2, cells in groups I-PP surrounding the agarose gel showed darkened stains. The difference in anti-PP activity was more significant on Day 6. Cells in group L-PP extended their cytoplasm to the gel and showed spotted light stain. In group I-PP, at least 10 layers of cells near the core of agarose were brown stained in their cytoplasm, indicating the anti-PP activity. In group H-PP, only the cells at the invasion front showed antibody activity.

### 3.3. Effect of Ascorbic Acid, DSP, and DSP/PP on Stimulating DSP-PP Transcript Expression

**Figure 4:**
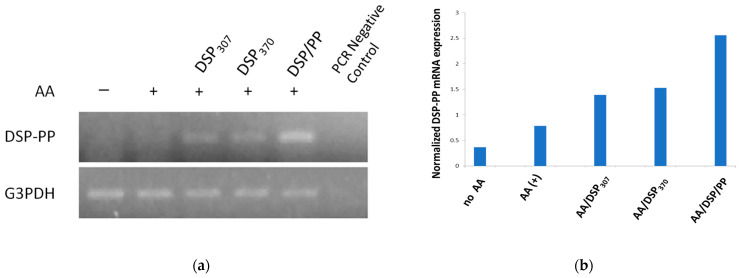
Effect of AA, DSP, and DSP/PP on promoting DSP-PP mRNA expression in M2H4 cells. (**a**) Representative agarose gel electrophoretic patterns of PCR product. (**b**) Normalized mRNA expression levels of M2H4 cells under different treatments.

As shown in Figure 4, AA promoted DSP-PP mRNA expression and DSP_307_ and DSP_370_ further enhanced the expression. DSP_430_/PP_240_ promoted highest level of DSP-PP mRNA expression. The relative DSP-PP mRNA expression (normalized with G3PDH mRNA expression) showed that recombinant proteins, especially DSP/PP, exhibited strong ability in promoting DSP-PP mRNA expression.

### 3.4. Inhibition of DSPP mRNA Expression by DSPP RNAi

**Figure 5:**
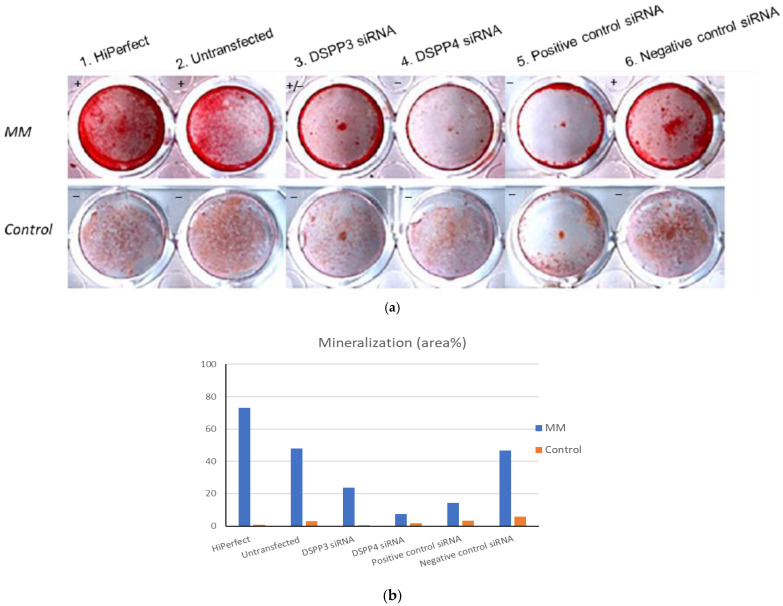
(**a**) Alizarin red stain of MDPC23 cell and mineralization: “+” represents strong mineralization; “−” represents no mineralization; “+/−” represents lower mineralization. (**b**) Area percentages of mineralization.

Figure 5 shows the mineralization states of extracellular matrixes (ECM) synthesized by MDPC23 cells cultured in either mineralization media (MM) or regular medium (Control). The ECM contains collagen type I (90%) and non-collagenous proteins (DSP and PP). In the MM panel, dental pulp cells treated with HiPerfect and untransfected cells (both without siRNA treatment) showed strong stains which indicated strong mineralization of ECM (shown as “+” in Figure 5a). Positive-control siRNA is known to affect mRNA expression. For cells treated with positive-control siRNA (sample 5), DSPP mRNA expression was affected and led no ECM mineralization (indicated as “−”). Contrarily, cells treated with negative-control siRNA (sample 6) presented unaffected mRNA expression and mineralization states. Cells treated with DSPP3 siRNA (sample 3) showed reduced ECM with weak stains (indicated as “+/−”). Cells treated with DSPP4 siRNA showed no stains (indicated as “−”).

In Panel Control, dental pulp cells in samples 1–6 were cultured in non-mineralization media. All samples showed no stains.

### 3.5. Effects of PP and DSP/PP on Pulp Regeneration in a Ferret Model

One ferret died before the euthanization date, and one restoration was lost before the euthanization. Accordingly, 51 tooth specimens were obtained.

**Figure 6:**
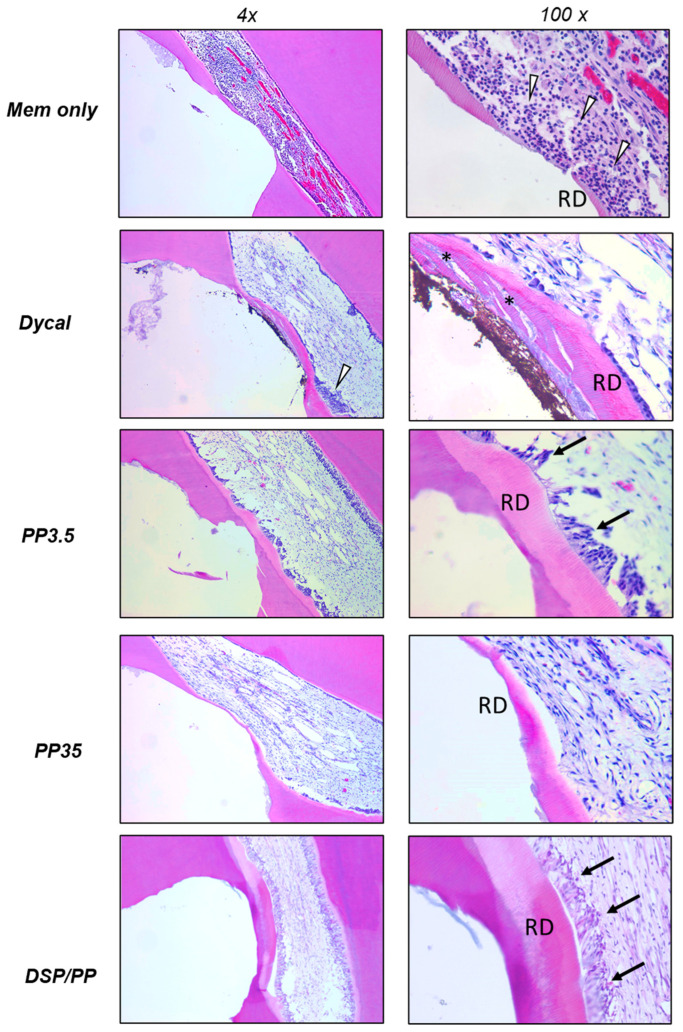
Histological findings of ferret teeth after direct pulp capping. Group Col showed pulp inflammation with infiltration of inflammatory cells (indicated by hollow arrowheads) and vessel congestion. The reparative dentin (RD) was thin and incomplete. Group Dycal also showed the presence of inflammatory cells. The reparative dentin was thick but contained tunnel defects (indicated by asterisks). Group PP3.5 presented reparative dentin with tubular structures and adjacent odontoblast lining (black arrows). In Group PP35, the reparative dentin was complete but thin. The odontoblast layer was absent. Group DSP/PP showed intact reparative dentin and odontoblast lining. Photos of left and right columns were at 4× and 100× magnification, respectively.

Most of the teeth capped with collagen membranes only (negative control) showed moderate-to-severe pulp inflammation with vessel congestion (Figure 6). The original odontoblast lining was destroyed and replaced by infiltration of inflammatory cells (polymorphonuclear neutrophils and mononuclear leucocytes). None of them (0%) showed complete reparative dentin formation. Group Dycal also showed damaged odontoblast lining and partial pulp tissue inflammation, such as infiltration of inflammatory cells and loose interstitial tissues. 

Despite of the inflammation, reparative dentin layer formation existed in nine (90%) teeth. However, these dentins appeared as atubular or exhibited tunnel defects. For teeth in group PP3.5, strong reparative dentin formation, healthy odontoblasts and dental pulp are found. 45.5% of specimens showed complete reparative dentin formation to seal the exposure site, and 36.4% appeared as regular tubular dentin. Teeth in group PP35 showed mild pulp inflammation, damaged odontoblast under the cavity, and partial reparative dentin formation. The teeth treated with DSP/PP showed healthy pulp core, viable dental pulp cells, and intact odontoblast lining. They also presented the thickest reparative dentin formation which already bridged the defects.

The scoring results of the histologic findings are summarized in Table 2. The statistic test showed significant difference in the aspects of inflammatory reaction, hard tissue formation, and quality of new dentin (*p* = 0.000, 0.002, 0.002, respectively). Groups DSP/PP and PP3.5 showed significantly less inflammation and more hard tissue formation compared to the other groups. For the quality of newly formed hard tissue, groups DSP/PP, PP3.5, and Dycal showed superiority compared to PP35.

## 4. Discussion

In this study, the effects of PP and the combined DSP/PP on the dental pulp cell responses and tissue reactions were evaluated by multiple methods. At first, the effects of different PP doses were examined by characterizing the proliferation and migration activity of treated MRPC-1 cells. Previous research has reported that PP works as the precursor of mineral aggregates and affiliates to collagen fibers to initiate dentinal mineralization [29]. The concentration-dependent effects of PP in the regulation of hydroxyapatite formation and growth interaction and the mineralization processes have been investigated with cell-free gel-precipitation methods [33]. PP of different concentrations play different role in either accelerating or inhibiting the hydroxyapatite formation at different stages [33,34]. On the other hand, our previous work established the agarose bead model and demonstrated that PP stimulated the chemotaxis behaviors and the synthesis of biomarkers of pulp cells after short-term culture [32].The present study also led the result that intermediate-dose (1 μg) PP was more effective in promoting the proliferation and migration of dental pulp cells. On Days 4 and 6, cells in the I-PP group intruded into the gel deeper than those in L-PP and H-PP groups did. High-dose PP only stimulated the cell migration at lower level.

PP dose may also influence cell morphology and differentiation. The MRPC-1 cell changed their morphology to the squamoid shape and specialized into highly active forms, with abundant granules and more cell division when they were in contact with the agarose bead or intruded into the gels. The phenomenon could be linked to the process of odontoblast differentiation. The cells surrounding the gels also show high PP expression after 6-day culture under the IHC assay. Accordingly, the PP dose also has significant effects on cell differentiation characteristics. However, significant expression of Col type I expression was not found on Day 6. In our previous work, strong Col type I expression was found in the day 2 culture of the intermediate PP dose group. These findings are consistent with the sequential matrix protein expression in tooth development process, in which Col type I expressed first followed by DSPP expression [30]. DSP-PP precursor protein was rapidly cleaved into DSP and PP, and PP may function as the nucleator of dentin mineralization. These results lent support that intermediate dose PP could be used to recruit dental pulp cells to the odontoblast layer and evokes the matrix gene expression during dentin formation and dentin repair.

In our previous study, the effect of combining AA and DSP treatments on the morphology of dental pulp M2H4 cells was shown [32]. When M2H4 cells were incubated in ascorbic acid with either recDSP_307_ or DSP/PP, more spread-out cells were observed in the cultures. Likely more ECM secreted and formed protruded edges. The present study further validated the effectiveness of these treatments in stimulating mRNA expression. After 8-day culture, lowest DSP-PP mRNA expression were shown in M2H4 cells of AA(−) group. Contrarily, AA induced DSP-PP mRNA expression in M2H4 cells. AA is known to induce cell differentiation in osteoblasts and odontoblasts. DSP-PP mRNA expression was associated with cell differentiation. M2H4 cells treated with recDSP_307_, recDSP_370_ and recDSP/PP proteins showed significantly increased DSP-PP mRNA expression, which corresponded with the morphologic findings of highly spread-out cells with active extracellular matrix formation in our previous study [32]. The increased DSP-PP mRNA expression was also associated with cell differentiation in regard to increased ECM synthesis, which was shown in the present mineralization assay.

Dental pulp MDPC23 cells have been recognized with their significant odontoblast makers including high DSP and PP expression, which undergo mineralization in appropriate condition [38]. Since DSP/PP proteins were suggested to participate in dental pulp cell differentiation into odontoblasts to synthesize ECM and resulted in cell mineralization. RNA interference (RNAi) was used to examine the role of DSP-PP mRNA expression during mineralization. In MM panel, MDPC23 cells under DSPP3 RNAi treatment (Figure 5a, sample 3) showed impaired mineralization in these cells and treatment with DSPP4 RNAi (sample 4) showed no mineralization. Thus, the RNAi experiments confirmed that the inhibition of DSP-PP mRNA expression affected dental pulp cell mineralization. These results suggest that DSP/PP plays an important role in inducing dentin matrix mineralization in vitro. DSP-PP mRNA transient expression in preameloblasts, then in a narrow time window, DSP-PP mRNA transient expression in both epithelial and mesenchymal cells finally sustained DSP-PP mRNA expression in odontoblasts. It is likely that the DSP-PP mRNA expression in epithelial cells could result in mature DSP and PP protein expression, which can induce dental pulp cell migration, differentiation, protein secretion and dentin mineralization. These findings lend strong support that DSP/PP has a role in promoting dental pulp differentiation, dentin matrix secretion and dental mineralization.

To test whether DSP/PP have a role in pulp tissue regeneration in vivo, here we used ferret animal models to test whether DSP and PP protein effect on recapturing developmental process such as promoting cell differentiation and forming reparative dentin. This model has been used by other investigators carrying out similar studies (i.e., demineralized dentin matrix and TGFb1) in reactionary and reparative dentin formation [25]. In the negative control group (Col), we observed serious dental pulp cell damage, inflammation, and little reparative dentin formation after 30 days. The collagen membrane did not have therapeutic effects, but dental pulp exhibited the natural repairing mechanism to provoke inflammation and reactional dentin. The severe inflammatory reaction may impair the regeneration of pulp tissue and dentin.

The calcium hydroxide-based capping material Dycal has been widely used in dental practices for decades. In this study, Dycal was used as a positive control. The teeth capped with Dycal showed both the inflammation reaction and dentine bridge formation. The pulp tissue was damaged, with infiltration of inflammatory cells and loose interstitial tissue. Although most of specimens exhibit the reactionary dentin formation, the dentine bridge was incomplete, likely with tunnel defects. The odontoblasts did not align well with the dentine bridge. The action mechanism of calcium hydroxide materials in pulp capping has been well investigated. Dycal exhibits high pH, which caused a series of repairing process comprising of inflammation and regeneration. Initially, the necrosis of a contact area of the pulp and an inflammation in the adjacent vital tissue occurred, followed by the formation of a dentine bridge at the border zone between the necrotic and vital tissue, and healing of the inflammation [8,41]. The initial inflammation may provoke the production of cytokine interleukin (IL)-1β, IL-6, dentin matrix protein 1, and DSP to induce the differentiation of odontoblast-like cells and induce dentine matrix formation [42]. The pulp inflammation has been considered as a prerequisite for the healing and regeneration of pulp receiving Dycal capping [43]. A previous study also suggests that mild inflammation is associated with thicker and more continuous dentine bridge formation [8]. However, Dycal-induced tissue reactions inevitably lead to severe consequences. The rhesus monkey animal study showed that most (89%) of the dentin bridges in display tunnel defects, failing to provide a seal against infection to the pulp [44]. It was reported that after 6 months most of the Ca(OH)_2_ capping material disintegrated and failed to establish complete dentine bridge [7,8,9]. Moreover, the initiated inflammatory reaction in the remaining pulp tissue may lead to severe and/or chronic detrimental effect to the pulp and eventually cause pulp necrosis [19,20,21].

Within the advancement of capping materials, the mineral trioxide aggregate (MTA) has been recommended to replace Dycal. MTA consists of a powder of fine trioxides and other hydrophilic particles. The hydration of the powder results in the formation of a colloidal gel with pH 12.5, which solidifies in a structure in about 3 to 4 h. The MTA has a mechanism similar to calcium hydroxide and therefore a powerful antibacterial agent. Biodentine is a two components material. The powder is mainly composed of tricalcium silicates. It also contains di-calcium silicate as a second core material and calcium carbonate and oxide as a filler. Biodentine is found to be associated with high pH 12 and releases calcium and silicon ions which stimulates mineralization and create “mineral infiltration zone” along dentin-cement interface imparting a better seal. The advantage of using Dycal, MTA and Biodentine is associated with high pH (12–12.5) to reduce bacterial infections. However, the bridge formation is not able completely seal the cavity [8].

In this study, two PP doses (3.5 μg and 35 μg) were designed to represent low and high doses. In group PP3.5, the specimens showed mild pulp inflammation response, favorable hard tissue formation including complete reparative dental bridge formation and tubular dentine type. An alignment of cells, some of which were elongated, polarized and odontoblast-like, was observed beneath the reparative dentine. On the other hand, group PP35 using PP at 10× dose, showed thin dentine bridge, and 27.3% of the teeth showed moderate to severe pulp inflammation. The odontoblast-like cells were absent underneath the reparative dentine. These results suggest that the lower PP dose promoted reparative dentin formation, maintained healthy dental pulp and healthy odontoblasts. PP has been well established as a nucleator during dentin matrix mineralization [27,28,45]. PP thus possesses chemotactic activity and can be used to recruit dental pulp cells to the odontoblast layer. The intermediate-PP dose has been approved to be more effective in promoting cell migration, proliferation, and differentiation, and mineralization [32]. Here, we further provided data that low native PP protein can promote reparative dentin formation in ferret dental cavity. The high-dose PP may limit cell proliferation and colonization, and thus result in inferior pulp regeneration ability. These results showed the amount of PP application is critical for proper reparative dentin formation.

The group using DSP/PP proteins resulted in the favorable healing response and hard tissue reaction in this study. The recombinant DSP/PP proteins promoted complete dentinal bridge formation, and strong reactionary dentin along the pulpal wall. Particularly, the application of DSP/PP proteins to exposed pulp showed viable dental pulp cells, no inflammation and intact odontoblast-like cell lining. This result supports that DSP/PP as the pulp capping material may stimulate the pulp regeneration and reactional dentin formation. Using recombinant PP or DSP/PP, the endogenous dental pulp cells were attracted to the damage site and to recapture the pulp cell differentiation and generate collagen type I and DSP/PP resulted in reparative dentin formation. Thus DSP/PP could potentially be used as a mimetic material for tooth repair and tissue engineering.

With these in vitro and in vivo findings, PP at certain dose, and DSP/PP may be useful for pulp tissue regeneration and dentin repair. Application of 3.5 μg native PP to pulp exposure site led to less inflammation and tubular reparative dentin formation. The application of DSP/PP recombinant proteins also lead to dental bridge formation with healthy odontoblasts and healthy dental pulp cells. PP and DSP/PP could potentially be used as a bio-mimetic material for tooth repair and tissue engineering. Our future works will be focused to determine the appropriate PP and DSP/PP protein concentrations for reparative dentin formation.

## 5. Conclusions

The in vitro study demonstrated that PP dose and DSP/PP had significant influences on modulating the migration, differentiation, and transcript expression of dental pulp cells. PP at the intermediate dose was more effective to stimulate cell migration, differentiation and expression of PP, compared to high-dose PP did. The recombinant DSP_307_, DSP_370_, and DSP/PP were responsible for the DSPP transcript expression. We provided evidence that RNA interference (RNAi) (i.e., to knock down DSPP expression) reduced mineralization in dental pulp cells. These findings lend strong support that DSPP has a role in dental pulp differentiation, dentin matrix secretion and dental mineralization. In the ferret experiment, the recombinant DSP/PP enhanced reparative dentin formation, evoked a strong reactionary dentin formation and also showed an integral odontoblast lining. Native rat PP at the low concentration also can promote reparative dentin formation. DSP/PP proteins can likely recapture the odontoblast developmental processes such as promoting cell migration from dental pulp toward preameloblasts expressing DSP-PP mRNA, reducing cell proliferation, and inducing cell differentiation to upregulate DSP-PP transcript expression, eventually leading to reparative dentin formation. These studies demonstrate the potential use of the native PP and the recombinant DSP/PP proteins as novel bio-inductive materials for pulp capping agents or further pulp regeneration therapy.

## Figures and Tables

**Table 1:** The scoring system used to evaluate the pulp response to direct pulp capping [39,40].

Grade	Inflammatory Reaction	Hard Tissue Formation	Quality of Dentin Formation in the Bridge
1	Absent or few inflammatory cells	Heavy: hard tissue deposition as complete and continuous dentin bridge	Regular pattern of tubules
2	Mild: inflammatory cells only next to dentin bridge or area of pulp exposition	Moderate: hard tissue formation as incomplete and discontinuous dentin bridge	Irregular pattern of tubules
3	Moderate: inflammatory cells are observed in the part of coronal pulp	Slight: a layer of scattered and foggy hard tissue deposition	No tubules present
4	Severe: all coronal pulp/more than two-thirds of the root canal pulp tissue is infiltrated or necrotic	No hard tissue deposition	

**Table 2:** Scoring of histological findings of pulps capped with different test materials.

Group	No. of Specimen	Inflammatory Reaction (%) ^1^				Hard Tissue Formation (%) ^2^				Quality of Dentin Formation in the Bridge (%) ^3^		
		1	2	3	4	1	2	3	4	1	2	3
Col	9	0.0	22.2	55.6	22.2	0.0	11.1	55.6	33.3	0.0	11.1	88.9
Dycal	10	0.0	20.0	60.0	20.0	10.0	30.0	50.0	10.0	40.0	40.0	20.0
PP3.5	11	36.4	54.5	9.1	0.0	45.5	27.3	18.2	9.1	36.4	36.4	27.3
PP35	11	0.0	72.7	18.2	9.1	0.0	45.5	36.4	18.2	18.2	36.4	45.5
DSP/PP	10	70.0	30.0	0.0	0.0	80.0	20.0	0.0	0.0	80.0	20.0	0.0

^1^*p* < 0.001. ^2^ *p* = 0.002. ^3^ *p* = 0.002.

## Data Availability

The data presented in this study are available on request from the corresponding author.
